# Are cancer patients with high depressive symptom levels able to manage these symptoms without professional care? The role of coping and social support

**DOI:** 10.1002/pon.5896

**Published:** 2022-02-12

**Authors:** Esmée A. Bickel, Joke Fleer, Adelita V. Ranchor, Maya J. Schroevers

**Affiliations:** ^1^ Department of Health Psychology University Medical Center Groningen University of Groningen Groningen The Netherlands

**Keywords:** cancer, coping, depressive symptoms, oncology, psycho‐oncology, social support

## Abstract

**Objective:**

Around 25% of cancer patients experiences depressive symptoms. However, the majority does not receive formal psychological care because patients often prefer managing symptoms alone or with informal social support. Previous research has shown that adaptive coping and social support can indeed be effective in managing relatively mild depressive symptoms. However, higher depressive symptom levels rarely improve without psychological treatment. This longitudinal study examined how and to what extent coping and social support are related to reductions in depressive symptoms in cancer patients with moderate to severe depressive symptoms.

**Methods:**

Respondents were diagnosed with cancer in the past five years, experienced high depressive symptom levels (PHQ‐9 ≥ 10) and were not receiving psychological care at baseline. We collected data with self‐report questionnaires (including PHQ‐9, brief COPE and Social Support List) at two assessments, taken three months apart.

**Results:**

Although depressive symptoms decreased significantly between baseline and follow‐up, the average level at follow‐up was still moderate to severe. Patients using less avoidant coping, specifically less substance use, were more likely to report a reduction of depressive symptoms. We found no significant beneficial effects of approach coping and social support (coping) on the course of depressive symptoms.

**Conclusions:**

A significant group of cancer patients with high levels of depressive symptoms do not seem able to effectively manage depressive symptoms by themselves, especially those more likely to avoid dealing with their symptoms. Cancer patients can be educated about avoidant coping and its possible detrimental effects, as well as being informed about possibilities of psychosocial services.

## 1 INTRODUCTION

Approximately 25% of all cancer patients suffers from depressive symptoms^1^, ^2^, ^3^ which can have negative consequences for patients' quality of life, disease management and longevity.^4^, ^5^ Interventions to reduce these symptoms have been developed and treatments such as cognitive behavioral therapy have proven to be effective.^6^, ^7^ Furthermore, guidelines were made to ensure cancer patients are screened for depressive symptoms and guided to appropriate care.^8^, ^9^ However, around 75% of cancer patients with increased levels of depressive symptoms does not utilize psychological care.^10^, ^11^

The main reason for low care uptake is not perceiving a need for psychological care and wanting to manage symptoms alone or with the help of friends and family.^12^, ^13^, ^14^ Previous research suggests that cancer patients with relatively low levels of depressive symptoms can effectively manage these symptoms themselves.^15^, ^16^, ^17^, ^18^ However, high levels of depressive symptoms rarely decrease without psychological treatment.^19^, ^20^ Meta‐analyses of randomized controlled trials consistently showed that high levels of depressive symptoms in control groups rarely decrease.^21^, ^22^ Taking into account that many cancer patients want to manage depressive symptoms themselves, the question arises: can cancer patients with high levels of depressive symptoms effectively manage and reduce these symptoms without the help of a professional? The aim of the current longitudinal study is to examine the course of depressive symptoms over time in cancer patients with moderate to severe depressive symptoms and the role of situational coping and perceived social support herein.

Previous studies and systematic reviews have examined the role of coping and social support in the course of depressive symptoms over time, in the general population as well as in cancer patients.^23^, ^24^ Approach emotion‐focused coping with cancer (e.g. positive reframing, acceptance), approach problem‐focused coping (e.g. problem‐solving) and high levels of social support have predicted decreases in depressive symptoms over time in cancer patients.^15^, ^16^, ^17^, ^18^ In contrast, avoidant coping with cancer (e.g. mental and behavioral disengagement) has been related to increases in symptoms over time.^25^

These previous studies have two main limitations: firstly, they included cancer patients with relatively low average depression levels (e.g. 14, 16, 25, and 26). Taking into account that managing depressive symptoms without the help of psychological treatment is especially difficult for cancer patients with high levels of depressive symptoms,^20^ these studies do not give information on how this group actually manages these symptoms. The current study is the first to only include cancer patients with high levels of depressive symptoms. Secondly, previous studies focused on the role of situational coping with *cancer,* rather than on coping with *depressive symptoms* in relation to the course of depressive symptoms over time. The transactional model of stress and coping emphasizes the importance of taking into account the specific situation when examining coping.^26^ Therefore, our study focused specifically on coping with depressive symptoms to get better insight into whether and how cancer patients can effectively manage their depressive symptoms. As far as we know, no other studies have looked into this yet.

This study aims to extend current literature by including a group of cancer patients with moderate to severe depressive symptoms to examine to what extent patients can decrease depressive symptoms themselves. We will investigate: (1) the course of cancer patients' depressive symptoms over a period of 3 months, and (2) the predictive role of coping strategies and social support on the course of depressive symptoms. We expect that, at the group level, levels of depressive symptoms will be rather stable over time. At the individual level, we expect variation in the course of depressive symptoms, with some people being able to reduce symptoms on their own and others not. We expect that use of approach coping and higher levels of social support will be related to a decrease in depressive symptoms, whilst the use of avoidant coping and lower levels of social support will be related to unchanged or increasing depressive symptoms.

## 2 METHODS

### 2.1 Study design

The data of this study are part of a larger project, focused on psychological care needs in cancer patients with depressive symptoms and has partly been used in an earlier paper.^27^ For this study, we used a longitudinal design with two online self‐report measuring moments, taken three months apart. The Medical Ethical Committee of the University Medical Center Groningen approved the study (2017/064).

### 2.2 Respondents

The study sample consisted of adult patients who were able to answer questionnaires in Dutch. Patients were included if they received a cancer diagnosis in the past five years and experienced moderate to severe levels of depressive symptoms (PHQ‐9 ≥ 10). Respondents who already received psychotherapeutic care were excluded from the sample.

### 2.3 Procedure

Kantar TNS – a large research agency with an extensive respondent panel (see https://www.kantar.com/) – recruited participants. They selected a group of possibly eligible respondents by screening for time since cancer diagnosis as well as age. This group was invited for study participation via e‐mail and screened for in‐ and exclusion criteria. Respondents started with the first questionnaire after receiving an information letter and providing informed consent to participate. Three months later, respondents were invited to fill in the second questionnaire.

### 2.4 Measures

#### 2.4.1 Demographic variables, cancer characteristics and psychological care

Demographic variables included gender, age, education, employment status and partner status. Cancer‐related variables included cancer type, cancer treatment, current treatment status (active/finished/planned) and time since diagnosis (in years). Uptake of psychological care between baseline and follow‐up (yes/no), previous psychological care (yes/no), current use of antidepressant medication (yes/no) and having a history of depression (yes/no) were included as possible control variables since these could have an impact on changes in depressive symptoms.^28^

#### 2.4.2 Depressive symptoms

Depressive symptoms were measured with the validated and widely used Patient Health Questionnaire (PHQ‐9) which measures DSM‐V symptoms of Major Depressive Disorder with nine items.^29^ Answering categories ranged from zero (not at all) to three (almost every day). Sum scores (sum of all nine items) of 10 or higher indicated moderate to severe depressive symptoms.

#### 2.4.3 Coping

Coping was measured with 14 two‐item subscales of the brief COPE.^30^ The questionnaire introduction included a summary of the depressive symptoms that respondents had indicated earlier (score of one or higher on a PHQ‐9 item) after which patients were asked what coping mechanisms they used when confronted with these symptoms. Answering categories ranged from one (I haven't been doing this at all) to four (I've been doing this a lot). The subscale scores were formed by summing the two corresponding items.

Similar to previous studies, we performed a principal component factor analysis of the coping subscales with Varimax rotation to condense the number of coping related variables.^31^ We found three factors: (1) approach coping (i.e. self‐distraction, active coping, positive reframing, humor, acceptance and religious coping, *α* = 0.66), (2) support seeking coping (i.e. seeking emotional and instrumental support and venting, *α* = 0.80), and (3) avoidant coping (i.e. substance use, behavioral disengagement and self‐blame, *α* = 0.67). The subscales planning and denial showed cross‐loading on two factors and were therefore excluded. The scores for the three coping factors consisted of the mean of the included items.

#### 2.4.4 Social support

Social support was measured in interactions and deficit with the Social Support List Interactions and Discrepancies (SSL‐I and SSL‐D)^32^ using the subscale ‘emotional support with problems’ (e.g., providing consolation, comfort, good advice). The social support interactions scale measured the frequency of social support interactions received and consisted of eight items with answering categories ranging from one (seldom or never) to four (very often) which were summed to create one score. Social support deficit measured patients' *perceptions* of the amount of social support they received. This was measured with eight items (similar to the interaction scale) with answering categories ranging from one (I miss it) to four (it happens too often), which were reversed and then summed to create one score for discrepancies. Cronbach's Alpha's in the current study were 0.88 and 0.84 respectively.

### 2.5 Statistical analyses

We performed our analyses with SPSS Statistics 25. For all variables, means and standard deviations or counts and percentages were calculated. We performed paired samples *t*‐tests to examine the course of depressive symptoms between baseline and follow‐up, both for the total score and for the separate symptoms, and calculated Cohen's D effect size to estimate the magnitude of change.^33^

To examine individual changes in depressive symptom levels, we calculated a change score by subtracting the total PHQ‐9 score at follow‐up from the total PHQ‐9 score at baseline. Previous research has shown that half the standard deviation is indicative of a clinically relevant change.^34^ We considered a change score lower than −0.5 SD to be a decrease in depressive symptoms and a score higher than 0.5 SD to be an increase in depressive symptoms. Scores that fell between −0.5 SD and 0.5 SD were considered to have remained stable.

We conducted bivariate and point‐biserial correlations to examine relations between demographic variables, cancer characteristics, variables related to psychological care, coping and social support on the one hand and depressive symptoms at follow‐up on the other hand. Next, we used linear regression analyses to examine the role of baseline coping and social support factors that significantly correlated with depressive symptoms at follow‐up, controlled for depressive symptoms at baseline and other factors that significantly correlated with depressive symptoms at follow‐up. We repeated these steps for the second regression analysis, to examine the predictive value of specific coping subscales. There was no multicollinearity between the variables in either of the regression analyses.

## 3 RESULTS

### 3.1 Participants

Out of the 2549 respondents approached for study participation, 469 respondents received a cancer diagnosis more than five years ago, 1491 respondents scored lower than 10 on the PHQ‐9, and 66 respondents were already receiving psychological care. Furthermore, 396 respondents did not reply or did not give consent to participate in the study. The remaining 127 respondents signed the informed consent form and completed the baseline questionnaire. In total, 107 respondents also filled in the follow‐up questionnaire and were included in the analyses. Patients who did not fill in the second assessment (*N* = 20) did not differ significantly in demographic variables and cancer characteristics from the group who did do so (*N* = 107). The flow‐chart with exact numbers of the overall screening procedure can be found in Figure S1. Patients' demographic and cancer characteristics are shown in Table 1.

**TABLE 1:** Demographic variables and cancer characteristics (*N* = 107)

Variable	*N* (%) or M ± SD
Gender (female)	62 (57.9%)
Age (in years)	60.9 ± 12.4
Education^a^	
Low	26 (24.3%)
Middle	48 (44.9%)
High	33 (30.8%)
Employment	
Retired	38 (35.5%)
Paid job	24 (22.4%)
Inability to work	23 (21.5%)
Homemaker	14 (13.1%)
Other^b^	8 (7.5%)
Partner status	
Married or registered partnership	70 (65.4%)
Single	17 (15.9%)
Divorced	5 (4.7%)
Other^c^	15 (14.0%)
Cancer type (multiple types possible)	
Breast	28 (26.2%)
Skin	23 (21.5%)
Male reproductive organs	14 (13.1%)
Digestive system	11 (10.3%)
Urinary tract	8 (7.5%)
Female reproductive organs	8 (7.5%)
Other^d^	20 (18.8%)
Cancer treatment (multiple treatments possible)	
Surgery	73 (68.2%)
Chemotherapy	36 (33.6%)
Radiotherapy	39 (36.4%)
Hormone therapy	28 (26.2%)
Immunotherapy	6 (5.6%)
Other	9 (8.4%)
Current treatment state	
Active	37 (34.6%)
Finished	55 (51.4%)
Planned	15 (14.0%)
Time since diagnosis (in years)	3.6 ± 1.4
Previous psychological care (yes)	57 (53.3%)
History of depression (yes)	33 (30.8%)
Care uptake between baseline and follow‐up (yes)	17 (15.9%)
Current use of antidepressant medication (yes)	13 (12.1%)

^a^ Low level education comprised no education, primary education, pre‐vocational secondary education, (basic and middle‐management), and secondary vocational education (level 1). Middle level education comprised pre‐vocational secondary education (combined and theoretical), secondary vocational education (levels 2, 3 or 4), senior general secondary education and pre‐university education. High level education comprised higher professional education and university/research‐oriented education (https://www.nuffic.nl/en/subjects/study‐holland/education‐in‐the‐netherlands).

^b^ Including searching for paid work and volunteering.

^c^ Including widow/widower, living together, divorced and having a partner but not living together.

^d^ Including respiratory tract, hematology, bone, endocrine, head/neck and central nervous system.

### 3.2 The course of depressive symptoms

The total level of depressive symptoms decreased significantly over time (see Table 2) with a medium and clinically relevant effect size (*d* = 0.48). However, the average depression score at follow‐up (*M* = 11.84, SD = 5.03) remained indicative of moderate to severe depressive symptoms (PHQ‐9 ≥ 10). Examining the course of distinct symptoms showed that loss of interest, depressed mood, sleep problems, fatigue and appetite change decreased significantly over time (see Table 2), with small to medium effect sizes. In contrast, low self‐esteem, concentration difficulties, psychomotor problems, and suicidality did not change significantly over time.

**TABLE 2:** Depressive symptoms at baseline and follow‐up (*N* = 107)

Depressive symptoms	Baseline Mean ± SD	Follow‐up Mean ± SD	Difference Mean ± SD	95% CI	Cohen's D
Total PHQ‐9 score	14.17 ± 3.69	11.84 ± 5.03	−2.33 ± 4.89**	[1.390, 3.264]	0.476
Loss of interest	1.88 ± 0.88	1.49 ± 0.85	−0.39 ± 0.92**	[0.216, 0.569]	0.427
Depressed mood	1.57 ± 0.79	1.23 ± 0.84	−0.34 ± 0.99**	[0.147, 0.526]	0.340
Sleep problems	2.22 ± 0.87	1.88 ± 0.98	−0.34 ± 1.12*	[0.123, 0.550]	0.302
Fatigue	2.51 ± 0.68	2.16 ± 0.85	−0.36 ± 0.86**	[0.190, 0.520]	0.413
Appetite change	1.79 ± 1.09	1.31 ± 1.03	−0.49 ± 1.13**	[0.270, 0.702]	0.431
Low self‐esteem	1.21 ± 1.05	1.02 ± 0.92	−0.19 ± 1.08	[−0.021, 0.394]	0.173
Concentration difficulty	1.38 ± 1.03	1.26 ± 0.97	−0.12 ± 1.05	[−0.080, 0.323]	0.115
Psychomotor	0.97 ± 1.00	0.84 ± 0.99	−0.13 ± 1.11	[−0.082, 0.343]	0.118
Suicidality	0.64 ± 0.90	0.65 ± 0.94	0.02 ± 0.86	[−0.183, 0.146]	−0.022

*Note*: Total PHQ‐9 scores ranged from 10 to 27, scores for separate items ranged from zero to three.

* Significant difference (*p* < 0.05) between baseline and follow‐up, ** Significant difference (*p* < 0.001) between baseline and follow‐up.

At an individual level, about half of the patients reported a decrease in depressive symptoms (*N* = 51, 47.7%). Yet, there were 40 patients (37.4%) who showed no clinically significant change in depressive symptoms and 16 patients (15.0%) who reported an increase in depressive symptoms. Some patients took up care between baseline and follow‐up, but this was not significantly correlated with changes in depressive symptom levels. At follow‐up, 64% of the patients still reported moderate to severe depressive symptoms (PHQ‐9 ≥ 10). The other 36% reported a decrease in symptoms to no to mild depressive symptoms at follow‐up.

### 3.3 The role of coping and social support

Bivariate correlations between coping strategies and social support at baseline and depressive symptoms at follow‐up showed that only avoidant coping was significantly and positively associated with depressive symptoms at follow‐up (see Table 3).

**TABLE 3:** Bivariate correlations between depressive symptoms at follow‐up, and coping as well as social support at baseline (*N* = 107)

	Mean ± SD	1	2	3	4	5	5a	5b	5c	6	7
1. Depressive symptoms baseline	14.17 ± 3.69	‐	0.405**	−0.249**	−0.010	0.194*	0.182	0.118	0.157	0.119	0.011
2. Depressive symptoms follow‐up	11.84 ± 5.03		‐	−0.085	0.061	0.408**	0.313**	0.282**	0.364**	0.111	−0.009
3. Approach coping	4.34 ± 0.83			‐	0.373**	0.002	−0.233*	0.065	0.046	0.345**	−0.236**
4. Support seeking coping	3.76 ± 1.23				‐	0.130	−0.050	0.218*	0.116	0.640**	−0.357**
5. Avoidant coping	3.31 ± 1.10					‐	0.775**	0.771**	0.784**	0.090	0.248*
a. Behavioral disengagement	3.55 ± 1.37						‐	0.335*	0.513**	−0.105	0.326**
b. Self‐blame	3.69 ± 1.61							‐	0.376**	0.137	0.257**
c. Substance use	2.69 ± 1.29								‐	0.173	−0.031
6. Social support interactions	17.35 ± 5.32									‐	−0.631**
7. Social support deficit	13.24 ± 4.20										‐

*Note*: Depressive symptoms ranged from 10 to 27, coping factors from two to eight, social support interactions from eight to 32 and social support deficit from eight to 24.

* Significant at *p* < 0.05, ** Significant at *p* < 0.01.

Next, we performed a regression analysis with avoidant coping as an independent variable and depressive symptoms at follow‐up as the dependent variable. This analysis was controlled for depressive symptoms at baseline (r [105] = 0.405, *p* < 0.001), and demographic variables and variables related to psychological care, that significantly correlated with depressive symptoms at follow‐up (see Table S1): care uptake (r_pb_ [105] = 0.233, *p* = 0.016), being divorced (r_pb_ [105] = 0.210, *p* = 0.030) and having a paid job (r_pb_ [105] = −0.202, *p* = 0.037). Results showed that avoidant coping was a significant predictor of the course of depressive symptoms (see Table 4). Using less avoidant coping at baseline was predictive of lower levels of depressive symptoms at follow‐up.

**TABLE 4:** Regression analyses outcomes regarding the impact of coping mechanisms on depressive symptoms at follow‐up (*N* = 107)

	B (SE)	95% CI	*β*	*t*	*p*
Regression analysis with avoidant coping					
Depressive symptoms baseline	0.426 ( 0.110)	[0.207, 0.644]	0.312***	3.868	<0.001
Avoidant coping	1.554 (0.386)	[0.787, 2.320]	0.341***	4.021	<0.001
Regression analysis with avoidant coping subscales					
Depressive symptoms baseline	0.424 (0.111)	[0.204, 0.644]	0.311***	3.828	<0.001
Behavioral disengagement	0.383 (0.350)	[−0.312, 1.079]	0.104	1.094	0.277
Self‐blame	0.366 (0.274)	[−0.177, 0.910]	0.117	1.338	0.184
Substance use	0.871 (0.390)	[0.097, 1.645]	0.223*	2.234	0.028

*Note*: Analyses were controlled for care uptake, being divorced, and having a paid job.

* Significant at *p* < 0.05, *** Significant at *p* < 0.001.

To expand our understanding of this effect of avoidant coping on the course of depressive symptoms, we examined each subscale of avoidant coping separately. Bivariate correlations (see Table 3) showed that all three subscales were significantly related to lower levels of depressive symptoms at follow‐up (i.e., using less behavioral disengagement (r [105] = 0.313, *p* = 0.001), less self‐blame (r [105] = 0.282, *p* = 0.003) and less substance use (r [105] = 0.364, *p* < 0.001)). Therefore, all three avoidant coping mechanisms were included in a second regression analysis (again controlling for depressive symptoms at baseline, care uptake, being divorced and having a paid job). Only substance use was significantly and positively related to the course of depressive symptoms (see Table 4). Patients who less strongly used substances as a way of coping reported a greater decrease in depressive symptoms.

## 4 DISCUSSION

We aimed to gain more insight into whether cancer patients can successfully manage moderate to severe depressive symptoms without professional care and the role of situational coping and social support herein. Although depressive symptoms decreased significantly over three months, the average level remained moderate to severe. Use of avoidant coping – specifically substance use – was significantly associated with the course of depressive symptoms. Cancer patients who used less avoidant coping (especially less alcohol or drugs) were more likely to report a decrease in depressive symptoms. Use of approach coping and social support had no significant role in depressive symptom reductions.

Despite a general decrease in depressive symptoms with a medium effect size, the average level of depressive symptoms at follow‐up was still moderate to severe. Results showed individual variation with about half of the patients reporting reductions in their depressive symptoms, and others reporting stable levels or even increases in depressive symptoms. At follow‐up, still two‐third of patients reported moderate to severe depressive symptoms. Overall, these results confirm previous literature and our hypothesis, suggesting that high levels of depressive symptoms in cancer patients often do not improve without formal treatment.^19^, ^20^, ^21^, ^22^ For distinct symptoms, patients mainly reported significant reductions in loss of interest and depressed mood, as well as reductions in more somatic problems, namely sleep problems, fatigue and appetite change. Low self‐esteem, concentration difficulty, psychomotor problems and suicidality did not decrease significantly, perhaps because their baseline values were already relatively low.

Use of avoidant coping (specifically use of alcohol and drugs) was significantly related to the course of depressive symptoms. Patients who used less avoidant coping strategies reported more reductions in their depressive symptoms than patients who used more avoidant coping. This is in line with previous research on coping with cancer which showed that using less avoidant coping was related to lower levels of depressive symptoms.^25^, ^35^, ^36^ It is important to inform patients who wish to manage depressive symptoms on their own about the signs and possible detrimental effects of using avoidant coping to help them manage these symptoms successfully.

Approach coping (i.e., acceptance, active coping, positive reframing) was only cross‐sectionally significantly related to depressive symptoms, in accordance with previous cross‐sectional research.^35^, ^36^ Contrary to what we expected, approach coping did not explain the course of depressive symptoms – something several previous studies did find.^17^, ^18^ However, other studies did not find an effect of approach coping – specifically active coping – on depressive symptoms three months later, which is similar to findings in our study.^37^, ^38^ Approach coping might thus not always be effective in managing depressive symptoms. Future research is needed to gain more insight into the time frame or conditions under which approach coping is effective in successfully managing high levels of depressive symptoms in cancer patients.

Also contrary to what we expected based on previous research,^15^, ^16^ social support related factors (i.e. support seeking coping, social support interactions and perceived deficit in social interactions) were not significantly associated with the course of depressive symptoms. A possible explanation is that the mere presence of supportive interactions is not sufficient: the ability to receive and accept support from others might be key in its effects on depressive symptoms. Perceiving more fear of receiving emotional support and perceiving a stronger impact of stigma have been shown to diminish the perception that others are available to offer support and can thereby increase depressive symptoms.^39^, ^40^ Future research could focus specifically on the (in‐)effectiveness of social support in managing high depressive symptom levels in cancer patients by including patients' ability to receive support.

Strengths of this longitudinal study are that we focused on patients who were experiencing moderate to severe depressive symptoms and that we measured coping with depressive symptoms specifically (rather than coping in general or with cancer). We tailored this question about the use of coping to the specific set of depressive symptoms that a patient had indicated at the beginning of the questionnaire.

### 4.1 Limitations

When interpreting our results, several limitations need to be taken into account. First, although we used a widely used and validated self‐report questionnaire to assess depressive symptoms that is strongly based on the DSM‐V criteria for a depressive disorder (i.e., PHQ‐9), a high score will not always accurately represent the existence of a depressive disorder. Therefore, results cannot be generalized to cancer patients with a formal depressive disorder. Previous research has concluded that the PHQ‐9 is a valid measure of depressive symptoms in cancer patients, even with the inclusion of the somatic symptoms that may overlap with symptoms of cancer and its treatment such as fatigue and sleep problems.^41^, ^42^ Secondly, we included a heterogeneous cancer sample in terms of time since diagnosis, type of cancer and phase in cancer treatment, with slightly more female patients, so results cannot be generalized to specific patient groups. Third, we only had two assessments with three months apart. It would be interesting to see whether the reduction in depressive symptoms is maintained, whether depressive symptoms decrease even more over a longer period of time, whether the effectiveness of certain coping strategies differs over time and whether there are subgroups in the course of depressive symptoms. Future research could therefore include a common starting point (such as prior to the start of medical treatment) and more assessments over a longer period of time to acquire a more accurate view of the course of depressive symptoms and the role of coping and social support herein.

### 4.2 Clinical implications

Although depressive symptoms in cancer patients decreased over time with an almost medium and thus clinically significant effect size, the average level of depressive symptoms remained above the cut‐off for moderate to severe depressive symptoms. This highlights the importance of increasing patients' awareness of the availability of psychological help and its importance in managing high levels of depressive symptoms. Patients who make no or little use of avoidant coping were most likely to successfully manage depressive symptoms. Cancer patients who are prone to using avoidant coping are less likely to decrease their depressive symptoms without professional support and might benefit from psychological care. Even though patients wish to manage depressive symptoms on their own or with support from friends and family, we found no significant benefits of approach coping and social support in the management of these symptoms.

## 5 CONCLUSIONS

In short, despite some improvement over time, the average level of depressive symptoms remained moderate to severe. For one‐third of cancer patients with high levels of depressive symptoms, their symptoms decreased to no or a mild level of symptoms. Specifically patients with strong tendencies to use avoidant coping experienced stable high levels or even an increase in depressive symptoms over time, and might benefit from professional psychological care.

## CONFLICT OF INTEREST

The authors have no potential conflicts of interest to report.

## Supporting information

Figure S1

Table S1

## Data Availability

The data that support the findings of this study are available from the corresponding author upon reasonable request.
