# Supplementary figures and images for: Are cancer patients with high depressive symptom levels able to manage these symptoms without professional care? The role of coping and social support
Source: Psychooncology. 2022 Feb 12;31(7):1102–9. doi: 10.1002/pon.5896 (PMC9542510; doi:10.1002/pon.5896)

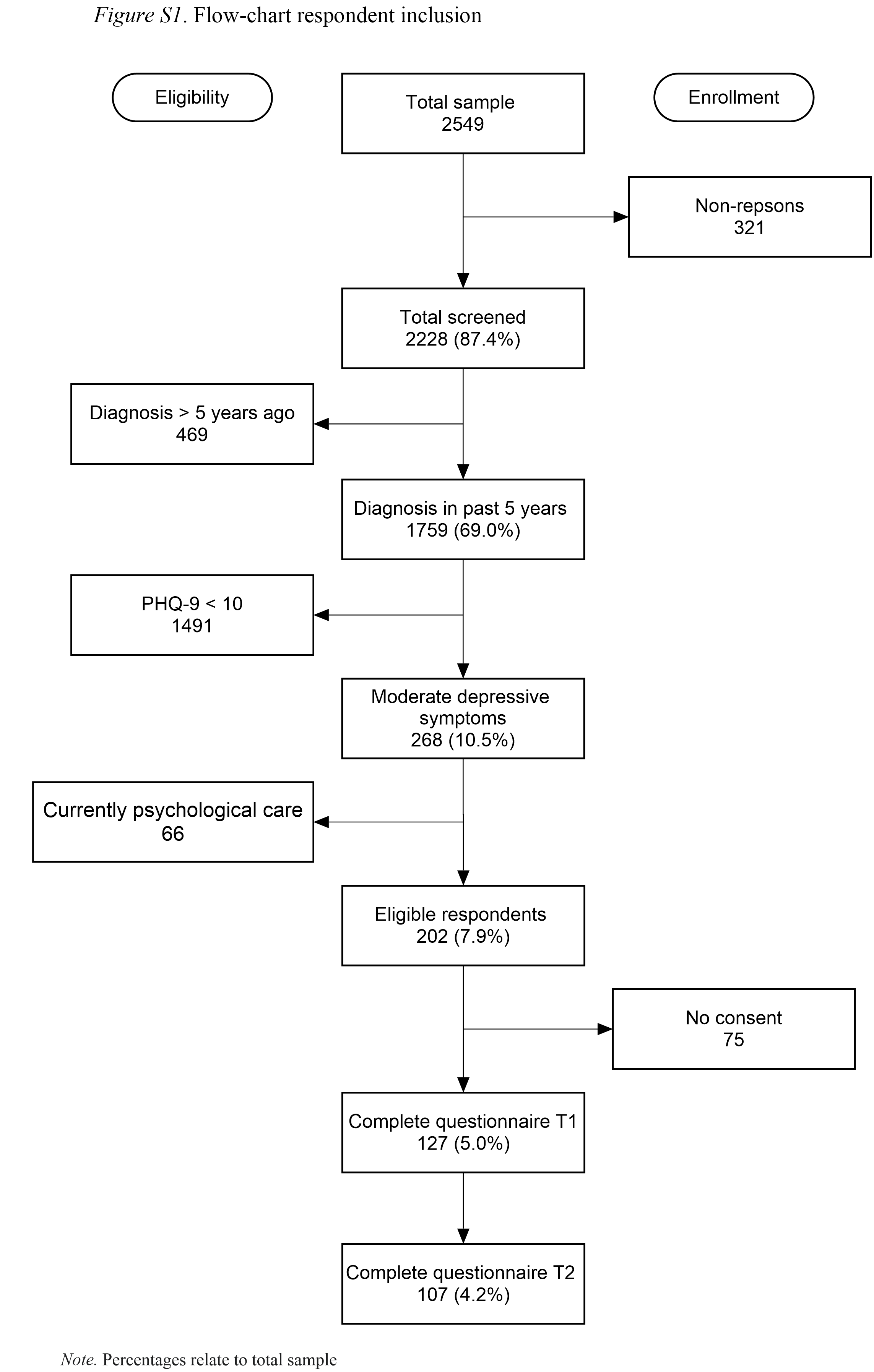

Supplement: Supplementary file 1 — Figure S1 [file PON-31-1102-s001.png]
