# Supplementary material for: Are cancer patients with high depressive symptom levels able to manage these symptoms without professional care? The role of coping and social support
Source: Psychooncology. 2022 Feb 12;31(7):1102–9. doi: 10.1002/pon.5896 (PMC9542510; doi:10.1002/pon.5896)
Supplement: Supplementary file 2 — Table S1 [file PON-31-1102-s002.docx]

Table S1. *Bivariate and point-biserial correlations*

|  | Depressive symptoms at follow-up |
| --- | --- |
| Control variables |  |
| Gender | .041 |
| Age | .187 |
| Education | -.048 |
| Employment |  |
| *Retired* | .027 |
| *Paid job* | -.202* |
| *Inability to work* | .144 |
| *Doing the household* | .057 |
| *Other* | n.s. |
| Partner status |  |
| *Married or registered partnership* | -.090 |
| *Single* | -.002 |
| *Divorced* | .210* |
| *Other* | n.s. |
| Cancer type |  |
| *Breast* | .083 |
| *Skin* | -.120 |
| *Male reproductive organs* | .040 |
| *Digestive system* | .103 |
| *Urinary tract* | -.012 |
| *Female reproductive organs* | -.126 |
| *Other* | n.s. |
| Cancer treatment |  |
| *Surgery* | -.110 |
| *Chemotherapy* | -.041 |
| *Radiotherapy* | .140 |
| *Hormone therapy* | -.036 |
| *Immunotherapy* | .024 |
| *Other* | .023 |
| Current treatment state |  |
| *Active* | -.020 |
| *Finished* | -.042 |
| *Planned* | .088 |
| Time since diagnosis | -.130 |
| Previous psychological care | -.045 |
| History of depression | -.159 |
| Care uptake | .233* |
|  |  |
| Coping |  |
| Approach | -.085 |
| Support seeking | .061 |
| Avoidant | .408** |
|  |  |
| Social support |  |
| Interactions | .111 |
| Deficit | -.009 |

* Significant correlation (p<.05) ** Significant correlation (p<.001)
